# TUBA1C is a Prognostic Marker in Low-grade Glioma and Correlates with Immune Cell Infiltration in the Tumor Microenvironment

**DOI:** 10.3389/fgene.2021.759953

**Published:** 2021-10-14

**Authors:** Hua Zhu, Xinyao Hu, Lijuan Gu, Zhihong Jian, Liqin Li, Siping Hu, Sheng Qiu, Xiaoxing Xiong

**Affiliations:** ^1^ Department of Neurosurgery, The Affiliated Huzhou Hospital, Zhejiang University School of Medicine (Huzhou Central Hospital), Huzhou, China; ^2^ Department of Neurosurgery, Renmin Hospital of Wuhan University, Wuhan, China; ^3^ Cancer Center, Renmin Hospital of Wuhan University, Wuhan, China; ^4^ Central Laboratory, Renmin Hospital of Wuhan University, Wuhan, China; ^5^ Department of Anesthesiology, The Affiliated Huzhou Hospital, Zhejiang University School of Medicine (Huzhou Central Hospital), Huzhou, China

**Keywords:** LGG, TUBA1C, prognosis, tumor microenvironment, tumor immune cell infiltration

## Abstract

TUBA1C, a microtubule component, contributes to the development of several cancers. Our purpose was to study the expression of TUBA1C, its potential prognostic value, and its effects on the infiltration of immune cells of low-grade glioma (LGG). Through applying multiple bioinformatics analyses, we extracted and analyzed datasets from TCGA, TIMER, GTEx, GEPIA, and HPA to investigate the potential oncogenic mechanisms of TUBA1C, including the correlation between TUBA1C and prognosis, immune-checkpoints, tumor microenvironment (TME), and infiltration of immune cells in LGG. GO functional annotations and KEGG pathway analyses were further applied to investigate the potential action of TUBA1C in LGG. We revealed that the mRNA levels of TUBA1C were increased in LGG tumor tissues than in normal tissues. Additionally, TUBA1C was up-regulated in the grade III of LGG than in grade II. Moreover, we found that TUBA1C may be an independent prognostic factor of LGG, and high TUBA1C expression correlated to a poor prognosis of LGG. TUBA1C expression was positively associated with the infiltration of B cells, CD8 T+ cells, CD4^+^ T cells, macrophages, dendritic cells, and neutrophils. TUBA1C was also verified to be co-expressed with immune-related genes and immune-checkpoints. GO and KEGG pathway analyses indicated that TUBA1C may potentially regulate the pathogenesis of LGG through immune-related pathways, including chemokine pathway; JAK-STAT pathway; natural killer cell mediated cytotoxicity; T cell receptor pathway; leukocyte migration; negative regulation of immune system process; regulation of lymphocyte activation; T cell activation and other pathways. In conclusion, TUBA1C expression is increased in LGG and high TUAB1C expression is related to a poor prognosis. TUBA1C may influence tumor development by regulating the tumor-infiltrating cells in the TME. TUBA1C may be a potential target for immunotherapy.

## Introduction

Glioma is a common and fatal tumor type in the central nervous system. Lower-grade glioma (LGG, WHO II, III) grow slowly with less malignancy and have superior survival outcomes compared with glioblastoma (GBM, WHO IV). Bioinformatics studies of gliomas have an important role in improving the accuracy of diagnosis and treatment, for example, thanks to bioinformatics, WHO have added molecular biomarkers such as isocitrate dehydrogenase mutation status to the diagnostic guidelines of glioma (Louis et al., 2016). Currently, although surgical resection of tumors combined with chemotherapy, radiotherapy, and neurorestorative therapy somewhat improves the prognosis of the patient, over 50% of LGG patients eventually develop into highly aggressive gliomas (Deng et al., 2019; Wang et al., 2020). Hence, new prognostic factors need to be identified for LGG.

Microtubules, which are assembled from highly conserved α/β-tubulin heterodimers (Bodakuntla et al., 2019), are one of the components of the backbone of eukaryotic cells, and exert a vital action in depolymerization and dynamic aggregation *via* cell division and replication (Kim et al., 2010). Previous studies have shown that α-tubulin participates in the development of several cancers, such as lung, prostate, and breast cancers (Boggs et al., 2015; Li et al., 2019; Zhang et al., 2016). Additionally, α-tubulin is involved in the occurrence of astrocytoma and chemoresistance in hepatocellular carcinoma (HCC) which greatly affects the prognosis of patients with liver cancer (Tsai et al., 2014; Hu et al., 2021). Notably, overexpression of TUBA1C, which is one of the α-tubulin subtypes is involved in the poor prognosis of HCC (Li et al., 2017), pancreatic ductal adenocarcinoma (Albahde et al., 2020), and lung cancer (Bian et al., 2021). However, whether TUBA1C can affect the prognosis of LGG has not been explored.

In recent years, immunotherapies have been applied for the treatment of glioma patients and have changed the treatment paradigm for glioma (Lim et al., 2018). Tumor-infiltrating immune cells (TIICs) are known to affect the immune system, processing abnormal biological behavior in a complex manner and exerting an essential action in response to immunotherapies (Xiong et al., 2018). In addition, genes associated with immune components of the TME are of great value as prognostic biomarkers (Le Rhun et al., 2019). Recently, several studies have proposed that TUBA1C is related to poor prognosis in HCC (Wang et al., 2017), pancreatic ductal adenocarcinoma (Albahde et al., 2020), and lung adenocarcinoma (Bian et al., 2021). However, no studies have yet explored whether TUBA1C overexpression affects the tumor immune microenvironment of LGG.

Through applying multiple bioinformatics analyses, we extracted and analyzed datasets from The Cancer Genome Atlas (TCGA), genotype-tissue expression (GTEx), Tumor Immune Estimation Resource (TIMER), Gene Expression Profiling Interactive Analysis (GEPIA), and the Human Protein Atlas (HPA) to explore the potential oncogenic mechanisms of TUBA1C, including correlations between TUBA1C and the prognosis, immune-checkpoints, TME and TIICs in LGG. Gene ontology (GO) and Kyoto Encyclopedia of Genes and Genomes (KEGG), pathway enrichment analyses were applied to investigate the potential functions of TUBA1C.

## Methods

### Data Source and Analysis of Differential Expressions

We downloaded cancer-related RNA sequences, clinicopathological, and survival data of LGG and GBM on the UCSC Xena web (https://xena.ucsc.edu/, originally from the TCGA). GTEx (http://commonfund.nih.gov/GTEx/) contains publicly available gene expression data from the RNA sequencing of 54 normal tissue sites from about 1,000 individuals (GTEx Consortium, 2013). Normal samples from the GTEx database and tumor samples from TCGA were applied to compare the differential expressions of TUBA1C between cancer and normal tissue. The TUBA1C expression data in TCGA (https://tcga.xenahubs.net) were extracted by using Perl software for subsequent analysis. The “wilcox.test” method was used to evaluate the differential mRNA expressions of TUBA1C in LGG. The cut-off was established as a False Discovery Rate (FDR) value <0.05. The “ggpubr” R package was used for the boxplot.

Gene Expression Profiling Interactive Analysis (GEPIA) (http://gepia.cancer-pku.cn/index .html) is a publicly available database developed by Peking University, China (Tang et al., 2017). To verify the differential TUBA1C expressions in LGG and normal tissues, we further used GEPIA to verify our results and constructed boxplot, and survival curves, such as overall survival (OS) curve and disease-free survival (DFS) curve.

### Immunohistochemistry (IHC) Staining

Immunohistochemical images of TUBA1C protein expression analyses, assessment of the differences in TUBA1C expression at the protein level, were performed in normal, LGG, and GBM tissues from the HPA (http://www.proteinatlas.org/). The anti-body HPA043684 was used for IHC.

### Identification of the Prognostic Factors for OS in LGG

Univariate and multivariate Cox regression analyses were used to assess TUBA1C and five major clinical and prognostic factors, including age, race, sex, grade, radiotherapy, to identify the proper terms to build the nomogram. The forest plots showed the HR, 95% CI, and *p*-value of each variable by using the “forestplot” R package. Using R software (version 4.0.2) with the packages “rms,” a nomogram was formulated according to the results of multivariate Cox proportional hazards analysis to predict the 1-yr, 3-yr, and 5-yr overall recurrence.

### Correlations Between TUBA1C and Clinicopathology or Survival in LGG

We extracted the survival information for each sample in the TCGA. We then selected several indicators: OS, disease-specific survival (DSS), DFS, and progression-free interval (PFS), to clarify the association of TUBA1C expression with the prognosis of LGG patients. The Kaplan-Meier (KM) and log-rank test were applied for survival analysis of LGG (*p* < 0.05) and survival curves were performed through R packages “survminer” and “survival.” Then, R packages “survival” was used for Cox analysis to identify the correlation of TUBA1C with survival. The R packages “ggpubr” and “limma” were applied for clinicopathological correlation analyses.

### Relationship Between TUBA1C and Immune Cells Infiltration in the TME of LGG

The TIMER (https://cistrome.shinyapps.io/timer/) database was applied to explore the correlations between LGG and TIICs. The TIICs in TIMER contained dendritic cells, B cells, neutrophils, CD4^+^ T cells, macrophages, and CD8^+^ T cells. Subsequently, we applied the ESTIMATE algorithm in the R package “estimate” and “limma” to calculate immune and stromal scores. We analyzed tumor purity and the infiltration of stromal/immune cells in the tissue of LGG based on TUBA1C expression data using CIBERSORT, which was developed to estimate the abundance of particular cells in hybrid cell populations applying gene expression datasets. We next analyzed the correlation of TUBA1C with TME or infiltration of immune cells by using R packages “ggplot2,” “ggpubr,” and “ggExtra” (cut-off: value of *p* < 0.01).

### Immune-checkpoint Analysis

SIGLEC15, TIGIT, CTLA4, CD274, HAVCR2, LAG3, PDCD1, and PDCD1LG2 were selected to be immune-checkpoint–relevant transcripts, and the expression data of these eight genes were extracted. R package “ggplot2,” “pheatmap” and “immuneeconv” were used to assess the expression of the immune-checkpoints and co-expression of TUBA1C with these immune-checkpoints. Potential immune checkpoint blockade response was predicted with the TIDE algorithm (Jiang et al., 2018).

### Co-Expression Analyses and Pathways Enrichment Analyses of TUBA1C in LGG

R packages “limma,” “reshape2” and “RColorBrewer” were applied to perform the co-expression analyses. GO and KEGG gene sets were from the Gene Set Enrichment Analysis website (https://www.gsea-msigdb.org/gsea/downloads.jsp). The GO functional annotations and the KEGG enrichment pathways analyses of TUBA1C were conducted using the R package “enrichplot2,” “clusterProfiler,” “limma,” and “org.Hs.eg.db.”

### Statistical Analysis

Gene expression data were normalized by log2-transformation. Normal and tumor tissues were compared using a two-group *t*-test. Kruskal-Wallis one-way ANOVA was used to make comparisons between groups greater than or equal to three. The Cox proportional hazards model, KM analyses, and log-rank test were conducted for all survival analyses. Spearman’s test or Pearson’s test was applied to analyze the correlation between two variables; *p* value <0.05 were considered significant. R software (version 4.0.2) was used for statistical analysis.

## Results

### The Different Expressions of TUBA1C in LGG, GBM, and Normal Tissues

**FIGURE 1:**
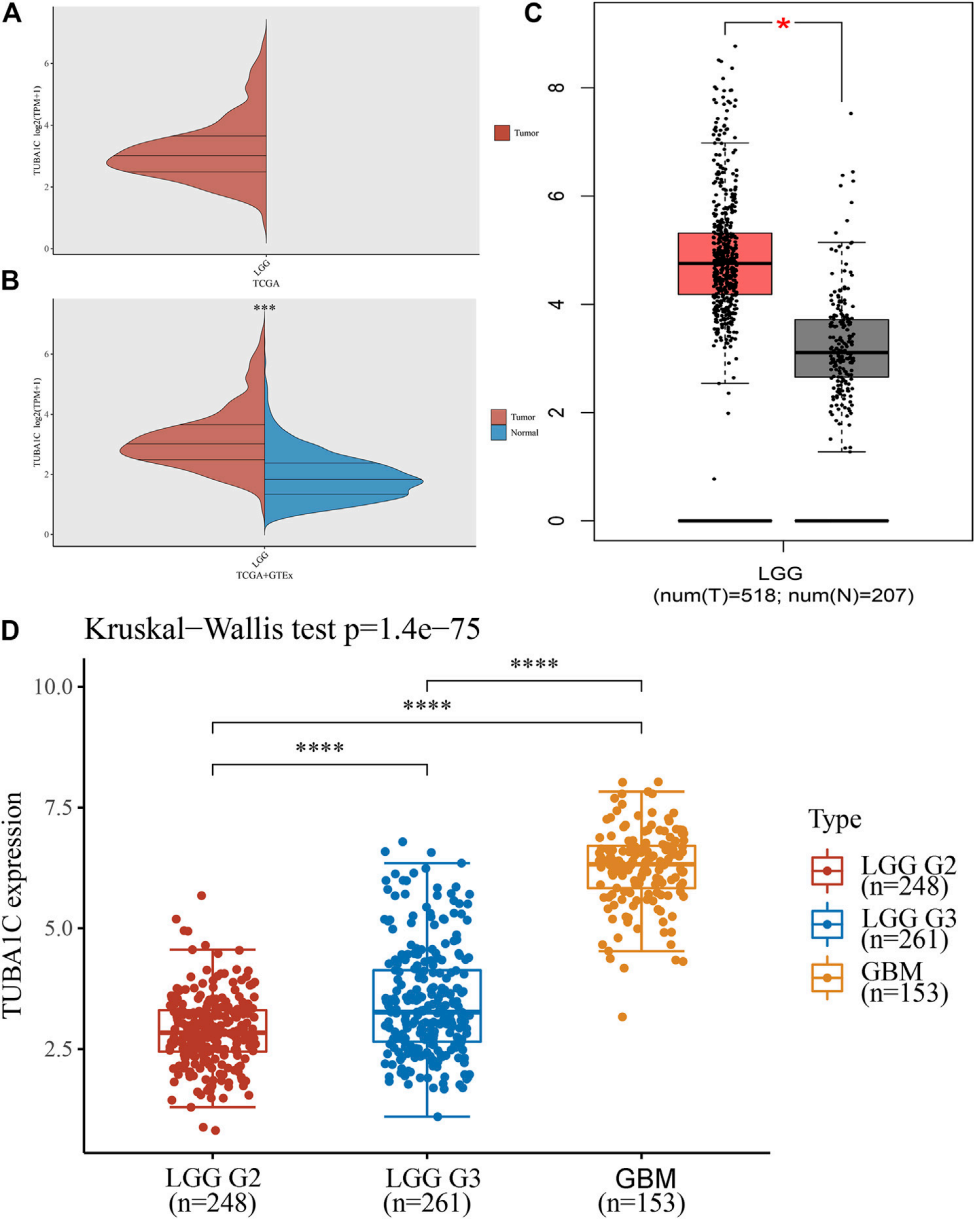
The different mRNA expressions of TUBA1C in LGG, GBM, and normal tissues. **(A**,**B)** mRNA expressions of TUBA1C in LGG and normal tissues. **(C)** mRNA expression of TUBA1C in LGG and normal tissues in the GEPIA. **(D)** Differential TUBA1C expressions in tumor stages in LGG and GBM. *p < 0.05, ***p < 0.001, ****p < 0.0001.

To evaluate the TUBA1C expression in LGG, GBM, and normal tissues, RNA sequencing data obtained from the TCGA were analyzed using R software. The TCGA profiles (including 509 LGG and 153 GBM samples) of mRNA expression were gained. Since the TCGA database lacked paracancerous tissue samples of LGG (Figure 1A), we downloaded 2642 normal samples from the GTEx database. We found that TUBA1C was overexpressed in LGG tumor tissues compared to the normal tissues (*p* < 0.001) (Figure 1B). We further verified the high TUBA1C expression in LGG tumor tissues in the GEPIA, the result indeed showed that TUBA1C was over-expressed in LGG than normal tissues (Figure 1C), which was consistent with our findings. Additionally, we evaluated the correlation between TUBA1C expression and tumor stage and found that the expression of TUBA1C was increased in stage III than stage II of LGG (*p* < 0.0001). Moreover, the expression of TUBA1C was elevated in GBM (WHO, stage IV) than stage III of LGG (*p* < 0.0001) (Figure 1D).

**FIGURE 2:**
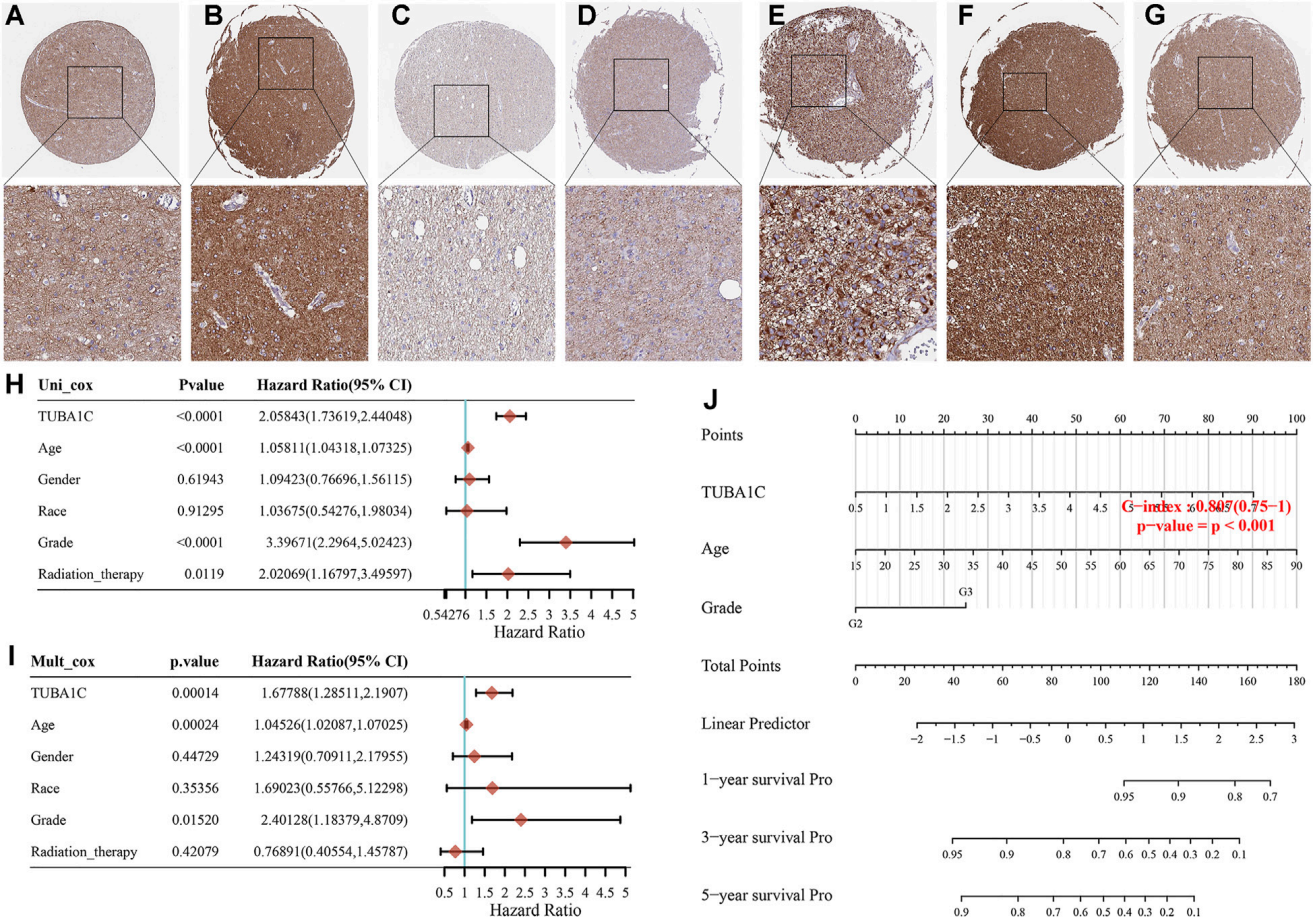
The TUBA1C expression detected by immunohistochemistry and the construction of prognostic signature in LGG. Representative immumohistochemical stainings in various normal **(A)**, LGG **(B**–**D)** and GBM **(E**–**G)** tissues. Hazard ratio and *p*-value of constituents involved in the univariable **(H)** and multivariate **(I)** Cox regression. **(J)** Nomogram consisting of risk score and other clinical indicators to predict the 1-yr, 3-yr and 5-yr OS of the patients with LGG.

To assess the expression of TUBA1C at the protein level, the IHC results were obtained and analyzed from the HPA. The results illustrated that the TUBA1C IHC staining was weak in the normal cerebral cortex (Figure 2A), whereas LGG tumor tissues had not detected the TUBA1C IHC staining or had high TUBA1C IHC staining (Figures 2B–D). However, the GBM tumor tissues had strong TUBA1C IHC staining (Figures 2E–G).

### Prognostic Value of TUBA1C in LGG

We further applied the univariable and multivariable Cox regression model to investigate the independent prognostic force of the signature. Univariable analysis results indicated that TUBA1C (HR = 2.05843, *p* < 0.0001), grade (HR = 3.39671, *p* < 0.0001), age (HR = 1.05811, *p* < 0.0001), and radiation therapy (HR = 2.02069, *p* = 0.0119) had prognostic value for the OS of LGG (Figure 2H). Similarly, in the subsequent multivariable stepwise cox regression analysis, TUBA1C (HR = 1.67788, *p* = 0.00014), age (HR = 1.04526, *p* = 0.00024), grade (HR = 2.40128, *p* = 0.0152) still maintained their prognostic values (Figure 2I). These results demonstrated that TUBA1C expression was an independent prognostic factor in LGG. Thereafter, TUBA1C, age, and grade were visualized in the nomogram. Nomograms of 1-yr, 3-yr, and 5-yr OS in the cohort are shown in Figure 2J.

**FIGURE 3:**
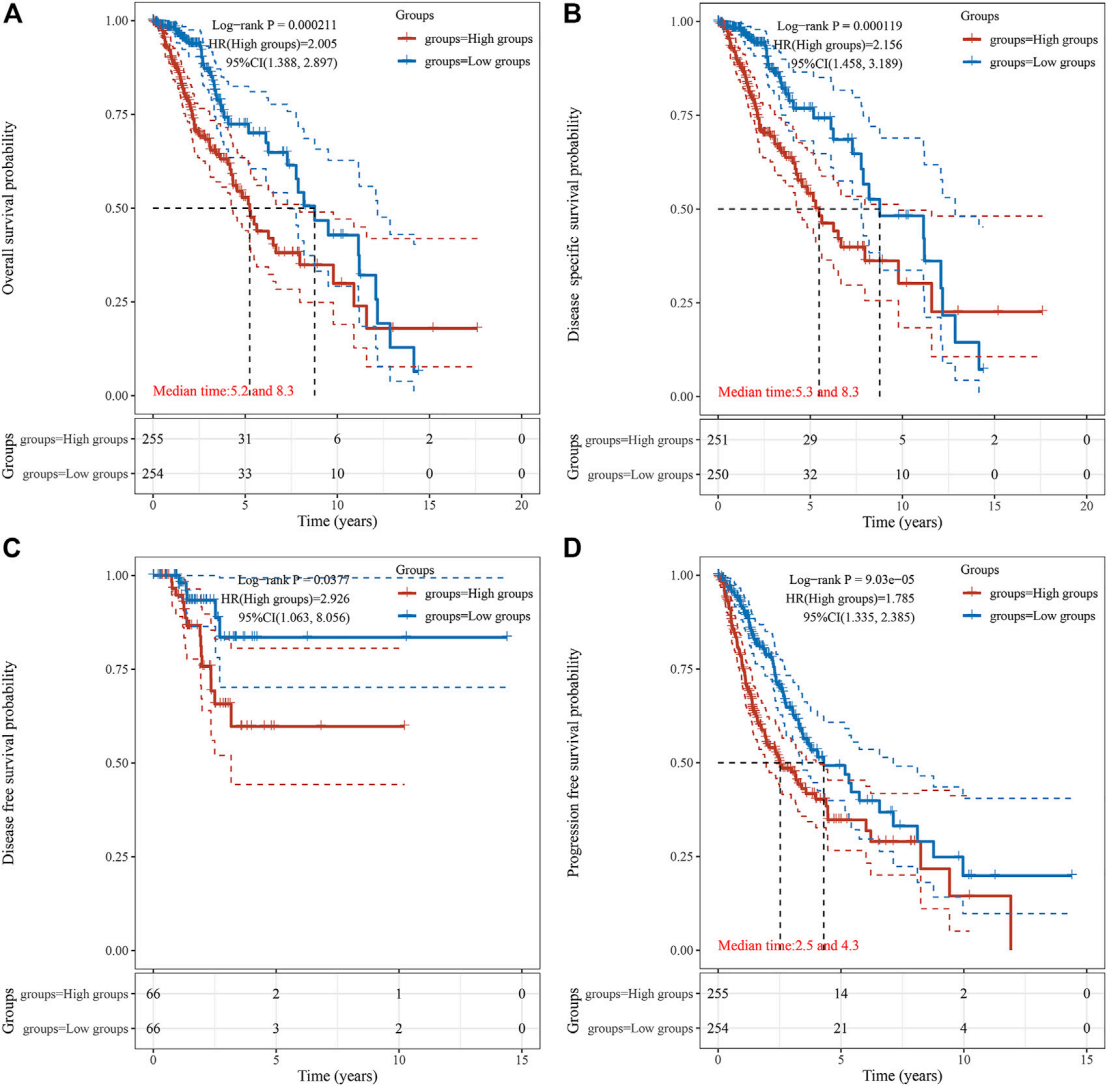
The correlation between TUBA1C expression and OS **(A)**, DSS **(B)**, DFS **(C)**, and PFS **(D)**. The correlation of TUBA1C expression with TME and TIICs.

Survival association analyses, including OS, DSS, DFS, and PFS were applied to further verify the correlation of TUBA1C expression with prognosis in LGG. KM plotter results showed that among the individuals with LGG (*p* = 0.0002) (Figure 3A), those with high TUBA1C expression had less time of survival. Furthermore, DSS analyses indicated a correlation between high TUBA1C expression and adverse outcomes in the LGG patients (*p* = 0.0001) (Figure 3B). Notably, LGG patients with high TUBA1C expression had poor DFS (*p* = 0.0377) (Figure 3C) and (*p* < 0.0001) PFS (Figure 3D). Furthermore, the OS and DFS curves were performed with the help of GEPIA. LGG patients with high TUBA1C expression had a poor OS (*p* = 0.00027) (Supplementary Figure S1A) and DFS (*p* = 0.00068) (Supplementary Figure S1B), which is consistent with our results in Figure 3.

**FIGURE 4:**
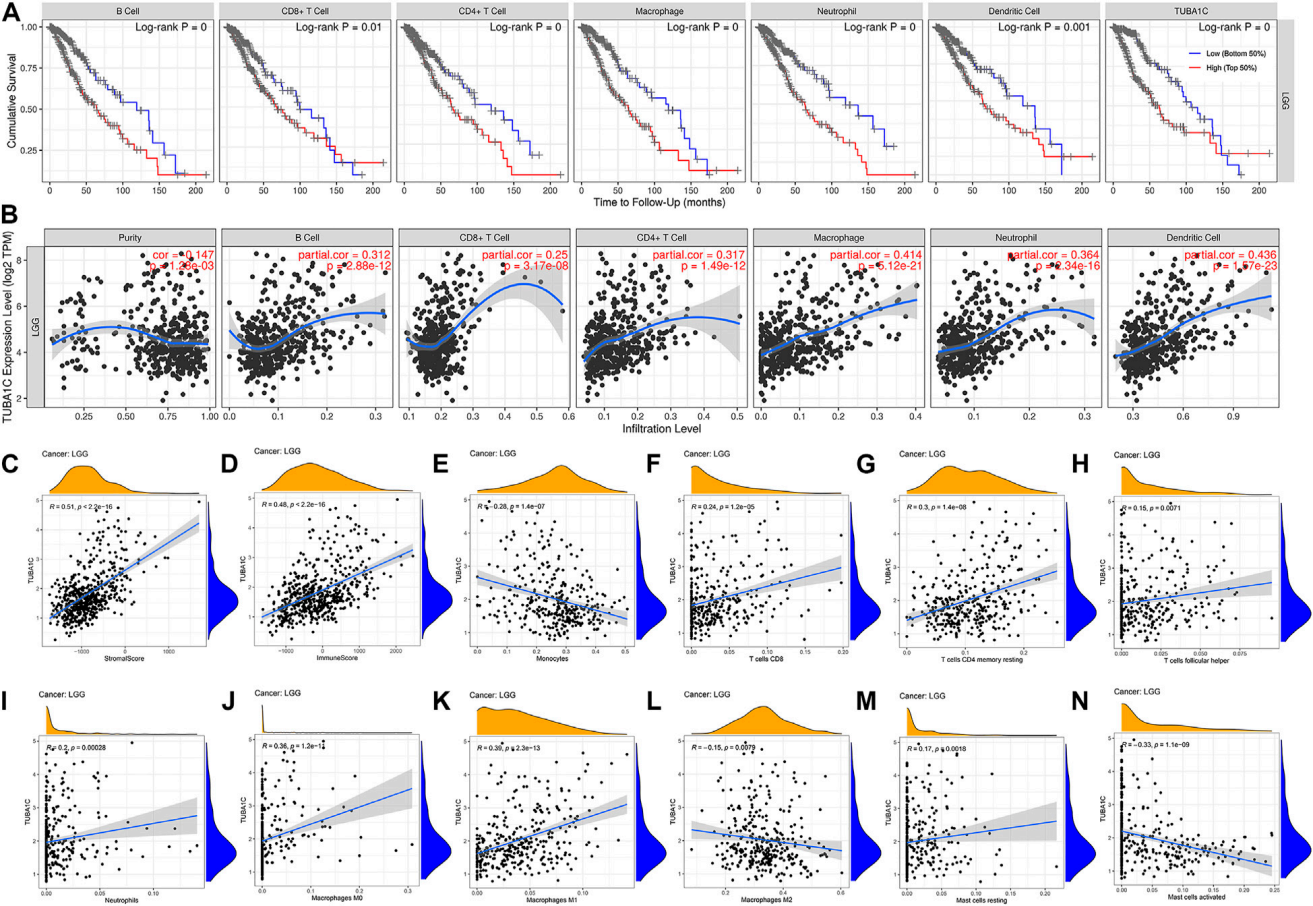
The correlations between TUBA1C expression and immune cell infiltration in the tumor microenvironment. **(A)** Correlation of infiltrating levels of immune cells with the cumulative survival of LGG. **(B**,**E**–**N)** Relationships between TUBA1C expression levels and immune cell infiltration in LGG. **(C)** Correlation of TUBA1C expression and stromal score. **(D)** Correlation of TUBA1C expression and immune score. TUBA1C co-expressed with immune-related genes and immune-checkpoints.

To investigate the mechanism by which high expression of TUBA1C leads to a worse prognosis, we applied the TIMER to reveal the correlation of TUBA1C with infiltrating levels of six immune cell subtypes, including dendritic cells, neutrophils, B cells, CD8 T+ cells, macrophages, and CD4^+^ T cells. As presented in Figure 4A, LGG patients with high infiltrating levels of these immune cells had a poor cumulative survival compared with low infiltration with these immune cells. Moreover, the TUBA1C expression was positively correlated with the infiltration of dendritic cells (Cor = 0.436, *p* = 1.57e−23), neutrophils (Cor = 0.364, *p* = 2.34e−16), B cells (Cor = 0.312, *p* = 2.88e−12), CD8 T+ cells (Cor = 0.25, *p* = 3.17e−08), macrophages (Cor = 0.414, *p* = 5.12e−21), and CD4^+^ T cells (Cor = 0.317, *p* = 1.49e−12). These results illustrate that LGG patients with high TUBA1C expression had a poor cumulative survival, which is consistent with the results in Figure 2. Additionally, ESTIMATE algorithm was applied to investigate stromal and immune scores for LGG and analyze the correlations of TUBA1C expression levels with these two scores. We revealed that in LGG, TUBA1C expression was significantly positively related to the stromal (R = 0.51, *p* < 2.2e−16) (Figure 4C) and immune scores (R = 0.48, *p* < 2.2e−16) (Figure 4D). To further evaluate the TIICs in the TME of LGG, we investigated the correlation of TUBA1C with the infiltration of 22 immune cell subtypes, including regulatory T cells, gamma delta T cells, follicular helper T cells, CD8^+^ T cells, CD4^+^ naïve T cells, CD4^+^ memory resting T cells, CD4^+^ memory activated T cells, neutrophils, resting NK cells, activated NK cells, resting myeloid dendritic cells, activated myeloid dendritic cells, monocyte, resting mast cells, activated mast cells, macrophage M0, M1, M2, eosinophil, plasma B cells, naïve B cells, memory B cells. The results further demonstrated that TUBA1C expression was positively related to the filtrating levels of CD8^+^ T cells (R = 0.24, *p* = 1.2e−05) (Figure 4F) and neutrophils (R = 0.2, *p* = 0.00028) (Figure 4I), which was in line with the results from the TIMER. Moreover, we revealed that the TUBA1C expression was positively associated with the infiltration of resting CD4 memory T cells (R = 0.3, *p* = 1.4e−08) (Figure 4G), follicular helper T cells (R = 0.15, *p* = 0.0071) (Figure 4H), macrophages M0 (R = 0.36, *p* = 1.2e−11) (Figure 4J), macrophages M1 (R = 0.39, *p* = 2.3e−13) (Figure 4K), resting mast cells (R = 0.17, *p* = 0.0018) (Figure 4M), while they were negatively related to the infiltration of macrophages M2 (R = −0.15, *p* = 0.0079) (Figure 4L) and activated mast cells (R = −0.33, *p* = 1.1e−09) (Figure 4N).

**FIGURE 5:**
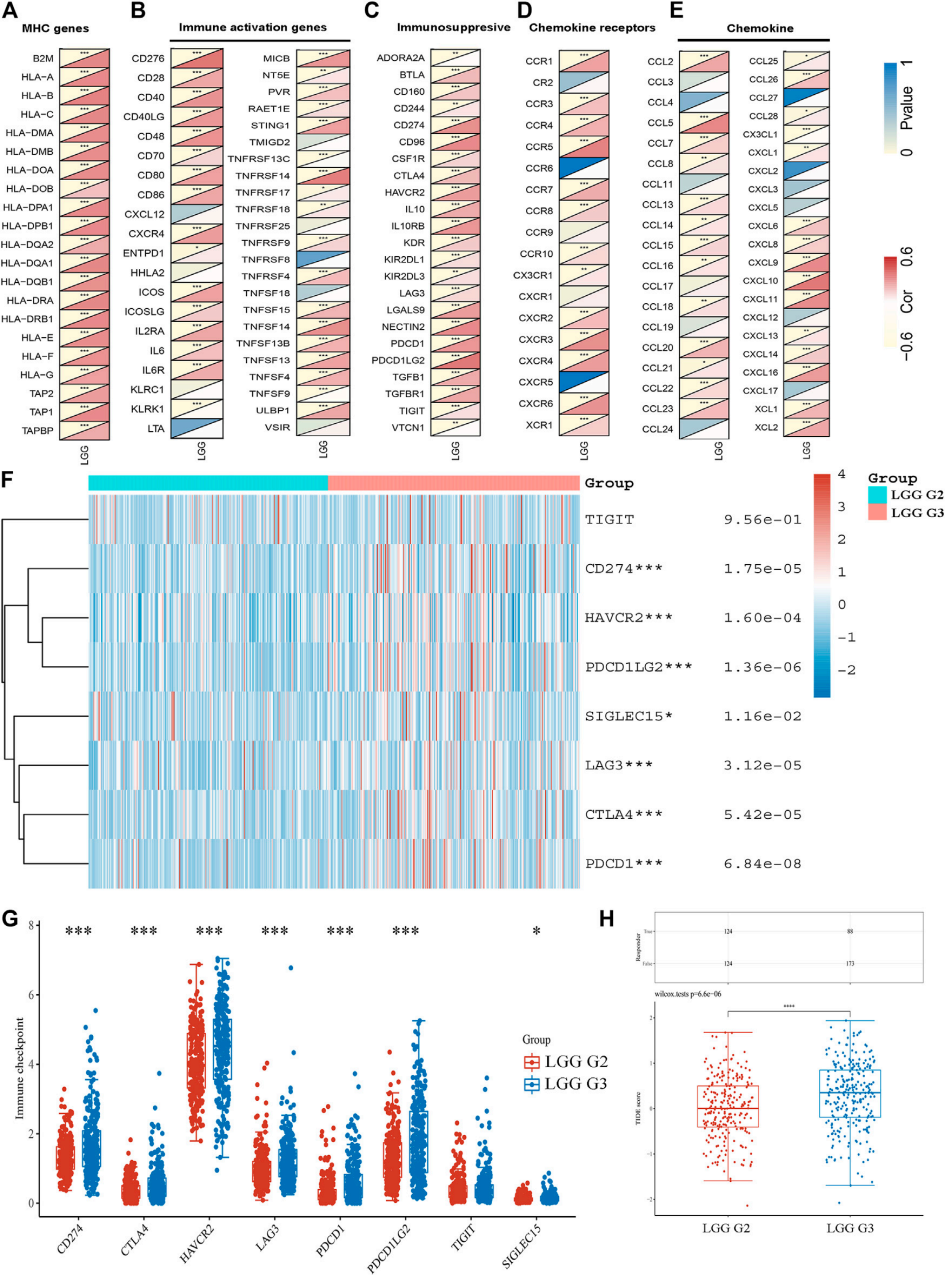
The co-expression of TUBA1C with immune-related genes and the expression of immune-checkpoints in LGG. Co-expression of TUBA1C with MHC genes **(A)**, immune activation genes **(B)**, immunosuppresive genes **(C)**, chemokine receptors related genes **(D)**, and chemokine related genes **(E)**. **(F**,**G)** Different expressions of immune-checkpoints in grade II and III of LGG. **(H)** Different responses to immune checkpoint blockade in grade II and III of LGG.

We further performed gene co-expression analysis to evaluate the mechanisms that TUBA1C was correlated to the infiltration of immune cells in LGG. MHC genes, immune activation genes, immunosuppresive, and chemokine (receptors) related genes were assessed. The results showed that TUBA1C positively co-expressed with all listed MHC genes (Figure 5A). TUBA1C had a positive relationship with most of the immune activation genes, such as genes that encoding CD276, CD28, IL6, CXCR4 (Figure 5B). Notably, almost all immunosuppresive genes positively co-expressed with TUBA1C (Figure 5C). Similarly, TUBA1C had a positive correlation with some chemokine (receptors) (Figures 5D,E).

The expression of immune-checkpoints, including SIGLEC15, TIGIT, CTLA4, CD274, HAVCR2, LAG3, PDCD1, and PDCD1LG2, were further investigated in the WHO grade II and III of LGG. The results illustrated that CD274 (*p* = 1.75e−05), HAVCR2 (*p* = 1.60e−05), PDCD1 (*p* = 6.84e−08), PDCD1LG2 (*p* = 1.36e−02), LAG3 (*p* = 3.12e−05), CTLA4 (*p* = 5.42e−05) and SIGLEC15 (*p* = 1.16e−02) were upregulated in WHO grade III compared with WHO grade II of LGG (Figures 5F,G). In addition, we revealed that LGG patients in WHO grade III had a better response to immune checkpoint blockade compared with LGG patients in WHO grade II (Figure 5H). Moreover, the results demonstrated that TUBA1C positively co-expressed with these immune-checkpoints (Table 1), indicating that TUBA1C may be a potential immunotherapy target.

**TABLE 1:** Correlation of TUBA1C with immune-checkpoint in LGG.

Genes	Cor	*p*-value
CD274	0.395	^a^/2.20e−116
CTLA4	0.242	^a^/3.21e−08
HAVCR2	0.461	^a^/2.20e−116
LAG3	0.216	^a^/9.25e−07
PDCD1	0.530	^a^/2.74e−38
PDCD1LG2	0.498	^a^/2.20e−116
TIGIT	0.290	^a^/2.86e−11
SIGLEC15	0.229	^a^/1.71e−07

a *p* < 0.001.

## Pathway Enriched Analyses of TUBA1C in LGG

**FIGURE 6:**
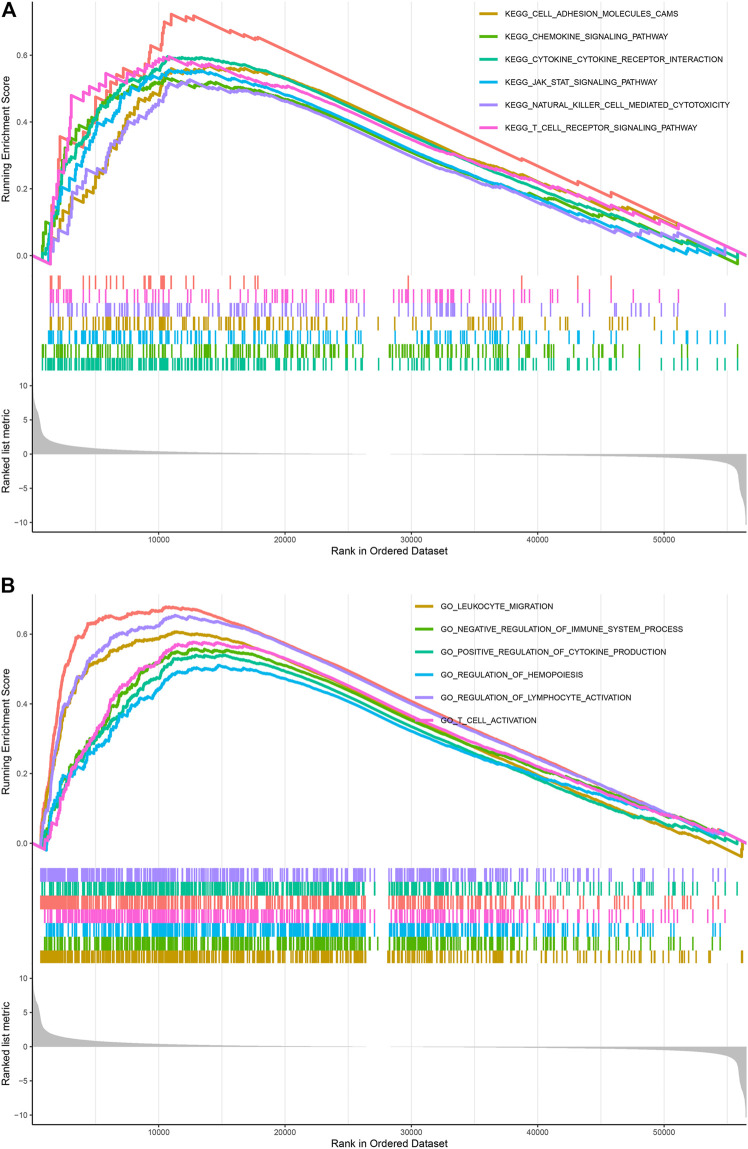
KEGG and GO pathways enriched analyses results. **(A)** Enrichment of pathways in KEGG with high TUBA1C expression. **(B)** Enrichment of pathways in GO with high TUBA1C expression.

GO functional annotations and KEGG pathway analyses were further applied to investigate the potential actions of TUBA1C in LGG. KEGG pathway analyses revealed six pathways that correlated with the high TUBA1C expression: cell adhesion molecules cams; chemokine pathway; JAK-STAT pathway; T cell receptor signaling pathway; cytokine cytokine receptor interaction; natural killer cell mediated cytotoxicity (Figure 6A). GO functional annotations showed the following six positively correlated pathways that correlated with the high TUBA1C expression: leukocyte migration; negative regulation of immune system process; positive regulation of cytokine production; regulation of hemopoiesis; regulation of lymphocyte activation; and T cell activation (Figure 6B). These findings demonstrated that TUBA1C was involved in immune-related pathways in the TME of LGG.

## Discussion

LGG is a prevalent primary malignant tumor of the central nervous system (Ostrom et al., 2013). Despite the fact that surgical resection combined with chemotherapy and radiotherapy can improve clinical outcomes, over 50% of LGG patients evolve to therapy-resistant high-grade aggressive glioma over time (Zhang et al., 2020). Therefore, we urgently need to identify new prognostic factors for LGG. The immune microenviroment plays a vital role in the development of tumors (Gajewski et al., 2013). Preclinical and clinical immunotherapies, including immune checkpoint inhibitors (ICI), active or passive immunotherapies, and gene therapies, have been used in gliomas (Barzon et al., 2006; Tan et al., 2017; Lim et al., 2018), further confirming that immunotherapies are essential for LGG treatment. Therefore, it is necessary to determine more potential immune-related prognostic biomarkers for immunotherapies.

It has been reported that TUBA1C, a multifunctional cytoskeleton protein, is a kind of α-tubulin subtype and that it is correlated with microtubules, participating in the progress of cellular mitosis and division (Jordan and Wilson, 2004; Albahde et al., 2020). It has been revealed that high the expression of TUBA1C predicts a poor prognosis of hepatocellular carcinoma (Wang et al., 2017), lung adenocarcinoma (Bian et al., 2021), breast cancer (Wang et al., 2019), and osteosarcomas (Li et al., 2010). However, the role of TUBA1C in LGG and whether TUBA1C has immune-related biological functions in LGG have not been reported. The present study is the first to discover that TUBA1C was elevated in LGG tumors compared to normal tissues (Figures 1B,C). In addition, the mRNA expression of TUBA1C in grade III was more increased than in grade II of LGG (Figure 1D). Moreover, our findings also indicate that the mRNA expression of TUBA1C in GBM was higher than in LGG (Figure 1D). High expression of TUBA1C was verified to be an independent prognostic factor of LGG (Figures 2H,I). We further found that high TUBA1C expression was related to poor survival in patients with LGG (Figure 3). These results demonstrated that TUBA1C may be served as a prognostic biomarker of LGG.

TME features have been verified to serve as biomarkers to evaluate tumor cell responses to immunotherapy and affect prognostic outcomes (Wu and Dai, 2017). Our results demonstrated the TUBA1C played an important role in cancer immunity. TUBA1C expression positively correlated with stromal and immune cell content in TME of LGG based on ESTIMATE score (Figures 4C,D). We further used the TIMER database to investigate whether the expression of TUBA1C correlated with the TIICs. The results demonstrated that TUBA1C was positively correlated with the infiltration of B cells, CD8 T+ cells, CD4^+^ T cells, macrophages, neutrophils, and dendritic cells (Figure 4B). Additionally, LGG patients with high infiltration of these immune cells had a poor cumulative survival compared with low infiltration with these immune cells. These findings indicate that high TUBA1C expression may be involved in poor survival through upregulating the infiltrating levels of B cells, CD8 T+ cells, CD4^+^ T cells, macrophages, neutrophils, and dendritic cells. Moreover, TUBA1C had different correlations with different immune cell subtypes, such as macrophages M0, M1, M2 (Figures 4J–L). Similarly, a previous study has illustrated that TUBA1C relates to the tumor-infiltrating cells of lung adenocarcinoma (Bian et al., 2021). We also demonstrated that TUBA1C was co-expressed with multiple immune-related genes, such as particular MHC genes, immune activation genes, immunosuppresive genes, chemokine receptor related genes, and chemokine related genes (Figures 5A–E). In addition, the results demonstrated that TUBA1C positively co-expressed with immune-checkpoints (Table 1), including CD274, CTLA4, HAVCR2, LAG3, PDCD1, PDCD1LG2, TIGIT, and SIGLEC15. Moreover, LGG patients in grade III had higher expression of these immune-checkpoints and better response to immune checkpoint blockade than LGG patients in grade II (Figures 5F–H). These results indicate that LGG patients with high expression of TUAB1C may have a better prognosis after immune checkpoint blockade treatment, further illustrating that TUBA1C may be a potential target for immunotherapy.

Moreover, the enrichment analyses showed that TUBA1C influences the tumor development process through multiple immune-related pathways, including cell adhesion molecules cams; chemokine pathway; JAK-STAT pathway; cytokine cytokine receptor interaction; T cell receptor pathway; natural killer cell mediated cytotoxicity; (Figure 6A); leukocyte migration; positive regulation of cytokine production; negative regulation of immune system process; regulation of lymphocyte activation; and T cell activation (Figure 6B). These results indicated that TUBA1C was involved in immune-related pathways in the TME of LGG.

In conclusion, our work demonstrated that TUBA1C expression is increased in LGG tumor tissues and high expression of TUAB1C is associated with a poor prognosis. TUBA1C may influence the tumor development process by regulating the tumor-infiltrating cells in the TME. TUBA1C may be a potential target for immunotherapy.

## Data Availability

The original contributions presented in the study are included in the article/Supplementary Material, further inquiries can be directed to the corresponding authors.
