# Supplementary material for: TUBA1C is a Prognostic Marker in Low-grade Glioma and Correlates with Immune Cell Infiltration in the Tumor Microenvironment
Source: Front Genet. 2021 Oct 14;12:759953. doi: 10.3389/fgene.2021.759953 (PMC8553001; doi:10.3389/fgene.2021.759953)
Supplement: Supplementary file 1 [file Table1.PDF]

Table 1. Correlation of TUBA1C with immune-checkpoint in LGG

**Genes**	**Cor**	***P*-value**
CD274	0.395	***/2.20e-116
CTLA4	0.242	***/3.21e-08
HAVCR2	0.461	***/2.20e-116
LAG3	0.216	***/9.25e-07
PDCD1	0.530	***/2.74e-38
PDCD1LG2	0.498	***/2.20e-116
TIGIT	0.290	***/2.86e-11
SIGLEC15	0.229	***/1.71e-07
