# Supplementary material for: TUBA1C is a Prognostic Marker in Low-grade Glioma and Correlates with Immune Cell Infiltration in the Tumor Microenvironment
Source: Front Genet. 2021 Oct 14;12:759953. doi: 10.3389/fgene.2021.759953 (PMC8553001; doi:10.3389/fgene.2021.759953)
Supplement: Supplementary file 3 [file DataSheet1.DOCX]

Raw data link: https://www.jianguoyun.com/p/Df1VbBQQgYfiCRiU2I0E
